# Collection of Simulated Data from a Thalamocortical Network Model

**DOI:** 10.1007/s12021-016-9319-4

**Published:** 2016-11-11

**Authors:** Helena Głąbska, Chaitanya Chintaluri, Daniel K. Wójcik

**Affiliations:** Department of Neurophysiology, Nencki Institute of Experimental Biology of Polish Academy of Sciences, Warsaw, Poland

**Keywords:** Data sharing, Extracellular potential, Local field potential, Simulated data, HDF5, Microelectrode array, Modelling, Current source density

## Abstract

A major challenge in experimental data analysis is the validation of analytical methods in a fully controlled scenario where the justification of the interpretation can be made directly and not just by plausibility. In some sciences, this could be a mathematical proof, yet biological systems usually do not satisfy assumptions of mathematical theorems. One solution is to use simulations of realistic models to generate ground truth data. In neuroscience, creating such data requires plausible models of neural activity, access to high performance computers, expertise and time to prepare and run the simulations, and to process the output. To facilitate such validation tests of analytical methods we provide rich data sets including intracellular voltage traces, transmembrane currents, morphologies, and spike times. Moreover, these data can be used to study the effects of different tissue models on the measurement. The data were generated using the largest publicly available multicompartmental model of thalamocortical network (Traub et al., *Journal of Neurophysiology, 93*(4), 2194–2232 (Traub et al. 2005)), with activity evoked by different thalamic stimuli.

## Introduction

The complexity of experimental protocols in neuroscience grows with technology. This enables more voluminous data collection and their analysis requires increasingly sophisticated approaches. Such methods of analysis remain speculative unless tested properly. Striving to extract knowledge from experimental data we are often forced to apply analytic methods beyond their proven applicability domains. For example, consider multielectrode recordings of extracellular electric potential which is a common method to investigate brain activity. Accurate interpretation of recorded signals is a challenging task, due to the complex relationship between electric field and the neuronal activity. The high frequency component of the extracellular signal is dominated by the spiking activity of neurons near the recording electrodes (multiunit activity, MUA), while the low frequency part^1^ (LFP) is believed to reflect mainly postsynaptic activity, although other non-synaptic events such as action potentials, calcium spikes, or glial activity, also affect this signal (Buzsáki et al. 2012).

In the analysis of extracellular potentials we may wish to use signal decomposition methods, such as principal or independent component analysis for signals coming from coupled neural populations (Di et al. 1990; Łęski et al. 2010; Makarov et al. 2010), or other more complex methods which take into account the physiology, such as laminar population analysis (LPA) (Einevoll et al. 2007; Głąbska et al. 2016). We may wish to localize the neural activity through reconstruction of current sources from the LFPs (Mitzdorf 1985; Pettersen et al. 2006; Łęski et al. 2007, 2011; Potworowski et al. 2012), or combine different methods in more complex protocols (Łęski et al. 2010; Głąbska et al. 2014), and so on.

The results of such an analysis of experimental data might be consistent with our expectations, common sense, and literature. But how can we be sure that they are not accidental? They may be a consequence of fortuitous selection of a problem where a confluence of factors makes our analysis plausible even though, in fact, it is incorrect. Or how can we tell which method gives the best results, for example, in spike sorting (Einevoll et al. 2012) or CSD analysis (Wójcik 2015)? To answer these questions we must properly validate the methods of analysis before their application to the data of interest (Denker et al. 2013). In mathematics we prove the applicability of a technique, however, in real world situations this is usually not feasible. We believe the correct approach is to use simulated *ground truth data* with models as close as possible to the systems being studied and including the models of measurement.

By saying *ground truth data* we imply that we have access to the complete state of the system for any simulated moment, that is, we can access any variable, such as membrane potential, transmembrane current passing through any channel type, complete set of spike times, etc. In neuroscience, so far, ground truth data were mostly considered in the context of spike sorting (Harris et al. 2000; Quian Quiroga et al. 2004; Gold et al. 2007). There one typically considers benchmark data consisting of a set of simulated or recorded extracellular potentials accompanied by independent information on spike trains, coming from a simulation or from an independent, more direct recording, such as intracellular or juxtacellular (Rossant et al. 2016; Neto et al. 2016). For a broader discussion of ground truth data in validation of spike sorting methods, calcium imaging, LFP and CSD data analysis, as well as for related concepts, see Denker et al. (2013).

If we require validation of every analytic protocol with the ground truth data we are faced with the task of building complex network models for systems of interest combined with models of different experimental modalities. Modeling the measurement has been a factor largely ignored in the literature, yet the fact that we are forced to make inference on the behavior of thousands or millions of cells from tens of extracellular potential recordings, in our view, requires building testable links through complex models between the system’s activity and its measurement. We may believe in population (field) models providing adequate representations of measurement but then again, how do we verify this postulate in the first place?

In practice, to model the extracellular potential or other measurement modalities using complex compartmental models we have two basic approaches. We may specify the model, define electrode positions, and compute the potential on the fly. This is the usual approach used, for example, by Gold et al. (2006), Lindėn et al. (2013), and Parasuram et al. (2016). The advantage is that one avoids extensive storage of compartmental data. The disadvantage is that if we need the potential at a new point we must repeat the whole simulation, which may be difficult if we run a complex network where each simulation takes hours of run-time. An alternative, which we follow here, is to record the complete state of the whole system throughout the duration of the simulation. The disadvantage is that of large storage demands, however, we can use such data post hoc to compute multiple measurement modalities, test different models of field propagation in tissue, etc, without the need to repeat the simulation. Especially if one wants to use such data as ground truth for validation of methods of data analysis from arbitrary multielectrode setups, clearly, one cannot a priori specify all possible setups. We thus believe that for this kind of applications the approach we advocate here is superior, or at least, a useful alternative. For such data to be truly useful they must be publicly available, well documented, citable, and easily accessible.

Generating ground truth data requires significant modeling experience, time to prepare, run and document the simulation, and access to high performance computers. This whole exercise is often impractical for someone who would just want to validate applicability of a specific method of data analysis and apply it to her experimental data. To facilitate validation and comparisons of different methods of data analysis here we present a collection of data generated using a thalamocortical network model based on Traub et al. (2005), which is the most comprehensive publicly available model of early sensory systems available at the time of writing. The data provided here were used to test a combination of kernel Current Source Density method with Independent Component Analysis (Głąbska et al. 2014), to study the propagation of electric fields in a cortical slice (Ness et al. 2015), and to validate the generalized Laminar Population Analysis method (Głąbska et al. 2016). The data provided here include intracellular voltage traces, transmembrane currents, spike times, and morphologies, which can be used to calculate different measurement modalities. We also provide a collection of scripts to to compute the extracellular potential at arbitrary electrode positions. We intend these data to serve as a proxy for experimental ground truth data and as benchmarks for validation and comparisons of different methods of neural data analysis.

These datasets are provided in the Neuroscience Simulation Data Format (NSDF) (Ray et al. 2016). NSDF is an Hierarchical Data Format version 5 (HDF5) subspecification (The HDF Group 1997), providing specific internal organization for neural simulations. We believe that providing data in a standardized format will further aid its scientific merit, as visualization tools and analytic methods can assume a common interface facilitating their generalization.

## Methods

### Thalamocortical Column Model

The data provided here were generated with a network model of a single cortical column receiving inputs from thalamic neurons based on the work by Traub et al. (2005). The model consists of 3560 multicompartment neurons in fourteen populations: twelve cortical populations from four cortical layers, and two thalamic neuron populations. The structure of the model is described in Table 1, see also Traub et al. (2005) and Głąbska et al. (2014). The original model was tuned to experimental data from the rat’s auditory cortex (in vitro) and the barrel cortex (in vivo) and provided in IBM Fortran (ModelDB, accession number 45539). To simulate extracellular potentials, where the placement of neural morphology in space is meaningful, we combined the versions in NEURON (ModelDB, accession number 82894) which was well parallelized, and the NeuroML version (ModelDB, accession number 127353) from which we took the 3D shapes of neurons. In defining the multi-compartmental models, we retained the specification from the NEURON version, where each section consisted of exactly one segment. Finally, we added mechanisms in every segment of every cell to facilitate tracking of the transmembrane currents which are essential to compute the extracellular potentials and made the necessary modifications to store these data on an IBM Blue Gene Q.

**Table 1:** Cell types used in the model, abbreviation used in the datafiles, numbers of sections per cell and numbers of cells per population for the full model, and the positions of their somas

Soma location	Population	Abbreviation	Sections per cell	Cells per population	Soma position (*μ* *m*)
	pyramidal regular spiking	pyrRS23	74	1000	
	pyramidal fast rythmic bursting	pyrFRB23	74	50	
Layer 2/3	superficial interneurons basket	bask23	59	90	0–400
	superficial interneurons axoaxonic	axax23	59	90	
	superficial interneurons low threshold spiking	LTS23	59	90	
Layer 4	spiny stellate	spinstel4	59	240	400–700
	pyramidal tufted intrinsic bursting	tuftIB5	61	800	
Layer 5	pyramidal tufted regular spiking	tuftRS5	61	200	700–1200
	deep interneurons basket	bask56	59	100	
Layer 5/6	deep interneurons axoaxonic	axax56	59	100	700–1700
	deep interneurons low threshold spiking	LTS56	59	100	
Layer 6	pyramidal nontufted regular spiking	nontuftRS6	50	500	1200–1700
Thalamus	thalamocortical relay	TCR	139	100	
Thalamus	nucleus reticularis	nRT	59	100	4900–5200

Note that in this model there is one segment per section. In most of the datasets (1–23 of Table 2), all the above 3560 cells were used. In some datasets (24–28 of Table 2), only 10 % of the cells from each population were used

The axonal gap junctions from the original Fortran model were turned off for two reasons. First, the NEURON implementation of the Traub’s model was not tested sufficiently with gap junctions which could have been a consequence of significantly greater simulation times. Second, we were unable to use active gap junctions in the variable time step simulations which are necessary for precise computation transmembrane currents when using NEURON 7.1 and 7.2 versions on an IBM Blue Gene Q.

#### Spatial Organization of the Network

The contribution to the extracellular potential from a current source is proportional to its amplitude and inversely proportional to the distance between the source and the electrode. Therefore, the spatial organization of the sources, in this case the positions of all the segments, is essential to compute the extracellular potentials. In the previous versions of the Traub’s model (ModelDB, accession numbers 45539, 82894, 127353), the spatial location of neurons was not specified. To allow computation of extracellular potential we placed the cells so that somas of a given population were distributed uniformly in cylinders of diameter 400 *μ*m and height corresponding to the vertical extent of the layers as described in Table 1.

### Simulations

The simulations were carried out with the NEURON simulator (Hines and Carnevale 1997) version 7.2 on a Blue Gene Q computer utilizing 512 cores. Random ectopic axonal action potentials were turned on Traub et al. (2005).

To achieve high precision of computation (monitored by tracking the sum of all the currents) we used the variable time step integration implemented with CVODE (Cohen and Hindmarsh 1996) in NEURON. The values thus obtained at variable time steps were linearly interpolated with a NEURON function with 0.1 ms time step and saved. In several datasets we used one tenth (in terms of the number of cells) of the model, henceforth referred to as the small model, to decrease the size of the data accumulated.

To bring layer 5 and layer 6 pyramidal cells to fire we injected a constant depolarization current of amplitude 1 nA (or 0.5 nA for the small model) to the layer 5 pyramidal cell somas and 0.75 nA (or 0.375 nA for the small model) to the layer 6 pyramidal cell somas (Table 3, Datasets 1–8, 17–28). Without such a depolarization these cells would remain silent even after prominent input. These values were consistent with the default NEURON version of this model and correspond to the parameter awake set to 1. (or to 0.5 for the small model). For comparison, we also provide datasets without this current injection (Table 2, Datasets 9–16).

In addition to the transmembrane currents and the membrane potentials from all the segments, we recorded spike times of all the cells. In some simulations of the small model we also recorded the contributions to the currents from different channel types (potassium, sodium, calcium) and other sources (through synapses GABA A, NMDA and AMPA; capacitive currents, passive currents). For details of channel mechanisms included in specific cells consult the original paper (Traub et al. 2005) and the provided code.

#### Stimuli

During the first 50 to 60 ms (in some cases even longer) the network exhibits a transient turn-on behavior following which the spiking activity settles down. We then stimulated the network with two types of stimuli: 1) a sinusoidal current injection to thalamocortical relay (TCR) cells (oscillations), or 2) a short constant current injection to TCR cells (pulse). The sinusoidal stimulus was used to study the properties of the network and to increase the diversity of the datasets, while the pulse stimulus emulates an evoked response in the network, e.g. a response of the rodent barrel cortex to deflection of a few whiskers. Further details are provided in Section “Datasets”.

#### Transmembrane Currents

Following the Kirchoff’s current law, the sum of all transmembrane currents in a cell must be zero. However, this does not hold true for contributions from individual channel types. This makes analysis of such contributions challenging, nevertheless, there is some interest in such analysis in the community (Reimann et al. 2013). To allow studies of contributions to extracellular potential from different partial currents (passive, active, synaptic, etc.) we tracked the capacitive and passive currents as well as the currents through every channel present in the Traub’s model (sodium, potassium, calcium currents, NMDA, AMPA, GABA A, anomalous rectifier currents, two types of low threshold T type currents Traub 2003; Traub et al. 2005), as well as steady bias and ectopic currents (Traub et al. 2005).

### Calculation of Extracellular Potential

**Fig. 1:**
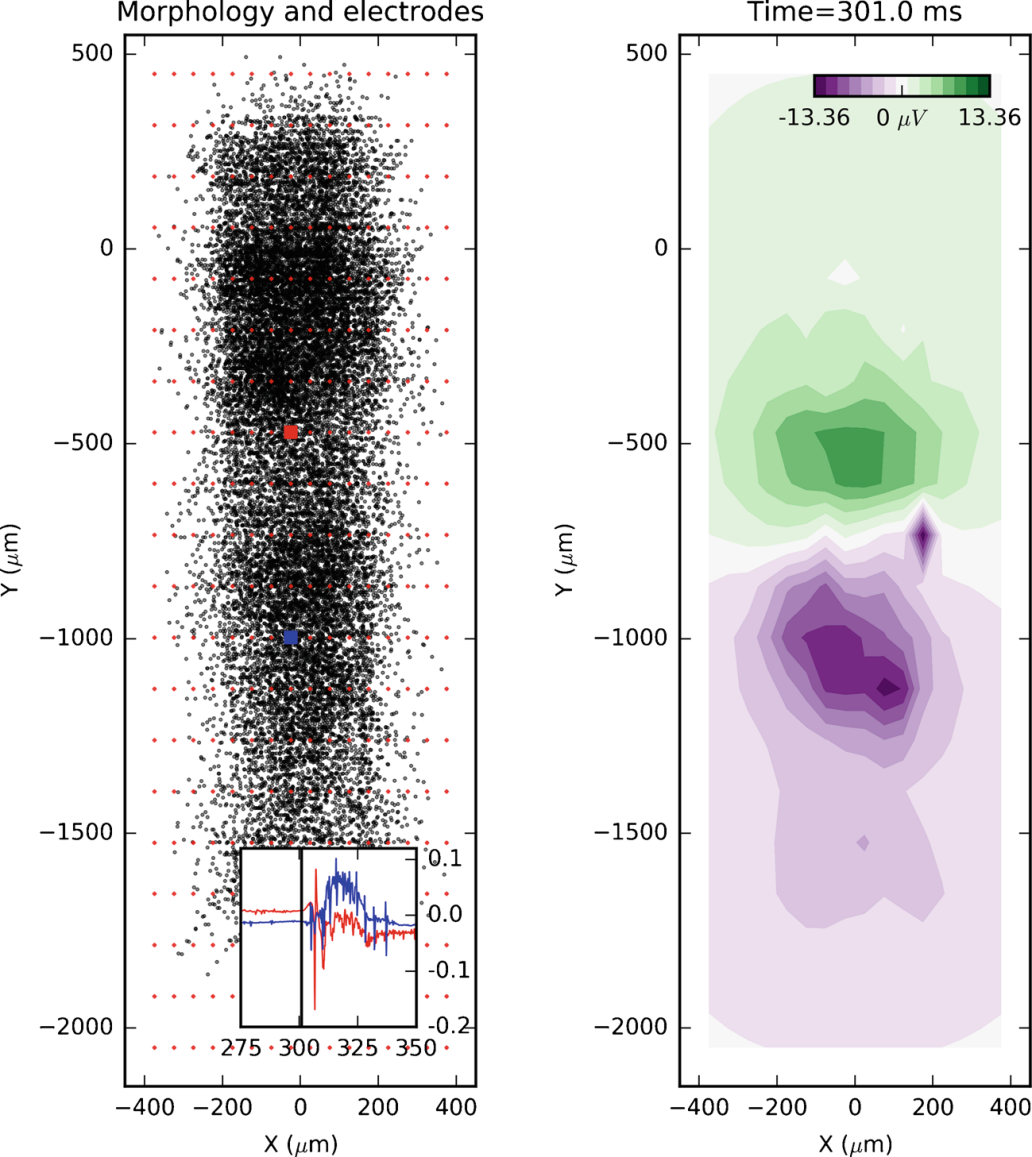
Here we show simulated 2D multielectrode recording of the extracellular potential generated by the studied network. On the *left*, the small red diamonds represent the electrode array (16 x 20) placed 25 *μ* *m* away from the cylindrical axis of the cortical column, while the *black circles* indicate the mid points of the segments of the cortical cells from the down-scaled network model (dataset 24). The inset figure is the extracellular potential recorded on two selected electrodes marked by *red* and *blue squares*. The *x* and *y* axis of the inset figure are time (ms), and potential (*m* *V*). On the right, we have linearly interpolated extracellular potential recorded at 301 ms from the onset of recorded simulation, which is indicated in the inset figure on the left plot with a *vertical black line*

Our main goal with these simulations was to provide a collection of datasets to validate different methods of analysis of extracellular recordings (LFP, multi-unit activity, spike trains). Since we cannot foresee specific arrangements of electrodes needed, specific bands to filter signals, models of field propagation, etc, we provide the recorded currents with a Python script to compute the extracellular potential at required positions. Figure 1 shows an example plot of extracellular potential in 2D plane spanned by a regular grid of 16 x 20 electrodes placed 25 *μ* *m* away from the cylindrical axis of the cortical column, as in a multielectrode array (MEA), at 301 ms after the start of recorded simulation, i.e., just after the onset of stimulus.

The script can be easily modified to indicate arbitrary electrode positions, use selected cortical cell populations, or even select specific currents to compute their contributions to the simulated recordings. For example, one can evaluate the contribution to the extracellular potential from capacitive currents of all pyramidal cells, etc. In these computations, we assume infinite homogeneous resistive extracellular medium recorded by ideal point electrodes and use the point source formula (Nunez and Srinivasan 2005):

1 $$ \varphi(\mathbf{x},t) = \frac{1}{4 \pi \sigma}{\sum}_{n=1}^{N} \frac{I_{n}(t)}{|\mathbf{x} - \mathbf{x_{n}}|}, $$

where *N* is the number of all the segments in the cortical part model, *I*_*n*_ is the transmembrane current from the *n*-th current source positioned at *x*_*n*_, *σ* is the extracellular conductivity. We assumed *σ*=0.3*S*/*m*. The point sources were placed at the centers of every segment. This script is provided only as a starting point for exploration. The users may want to consider more complex models of extracellular potential computation, such as the line source model for LFP (Holt and Koch 1999), or more complex models of tissue, such as a cortical slice in a multielectrode array dish (Ness et al. 2015), or frequency dependence of field propagation (Gomes et al. 2016), and the provided data could still be used.

### Sample Scripts Accompanying the Data

To show how to access the data we provide several example scripts in the Github repository https://github.com/Neuroinflab/Thalamocortical. In folder figures we provide scripts to generate the figures from this manuscript. Here, in the folder analysis_scripts we provide four scripts performing several basic tasks. These scripts are specific to the presented data but they can be easily extended to generic NSDF files.

#### lfp_parameters.py

Here you select the dataset to be used, 1D, 2D, or 3D geometry of electrode setups probing the field generated by the cortical column, and the cell populations and the model size to be used for computing the LFP in the next steps. The default parameters used by the rest of the files are set here.

#### calc_lfp.py

Computes the extracellular potentials using the transmembrane currents from the selected populations of cortical cells. The electrode positions and the populations to be used are taken from lfp_parameters.py file. We also provide here a function to convert the LFP calculated here into a NEO object list (Garcia et al. 2014), which can be used, e.g., in elephant (http://neuralensemble.org/elephant/) or other compatible software.

In this script we use the point source approximation, i.e., the segments are treated as point sources placed at the mid point of the segments. We also provide here the low pass filter function we used to compute the LFP from extracellular potentials (2nd order Butterworth filter).

#### create_plot.py

Plots the measured potentials for 1D and 2D electrode setups. It also shows the midpoints of the segments used in the LFP computation, marks the electrode positions, and shows interpolated potentials recorded using the 1D and 2D probes. The plot displayed for 1D case (laminar probe) shows potentials (*y* axis) versus time (*x* axis), and for the 2D case it shows interpolated potential in the plane of simulated MEA at a selected time point.

#### raster_spikes.py

Shows the raster plot for the whole network. The different colors represent different neuron types. Up arrow indicates an excitatory neuron. Down represents an inhibitory neuron.

These scripts do not constitute a toolbox although they may grow in the future. We provide these scripts to facilitate the uptake of the data provided here and implementation of other models of measurement. For more information read the provided Readme file and the scripts.

### File Format

The datasets are available in the Neuroscience Simulation Data Format (Ray et al. 2016), version 1.0. NSDF is a subspecification of Hierarchical Data Format version 5 (The HDF Group 1997), which introduces an organization of data within HDF5 in a way useful for storing the results of neuroscience simulations. HDF5 itself was developed particularly for storing scientific data, it is flexible, hierarchical, self describing, and allows efficient reading, writing, and storage of data. According to the NSDF specification the data must include some essential information about the simulation as attributes. These include units, start time, and time step of the simulation, units of the time step etc. Additionally, NSDF datasets must have meta-data attributes, which include the software used, the methods used, the name of the creator of the dataset, license and so on.

**Fig. 2:**
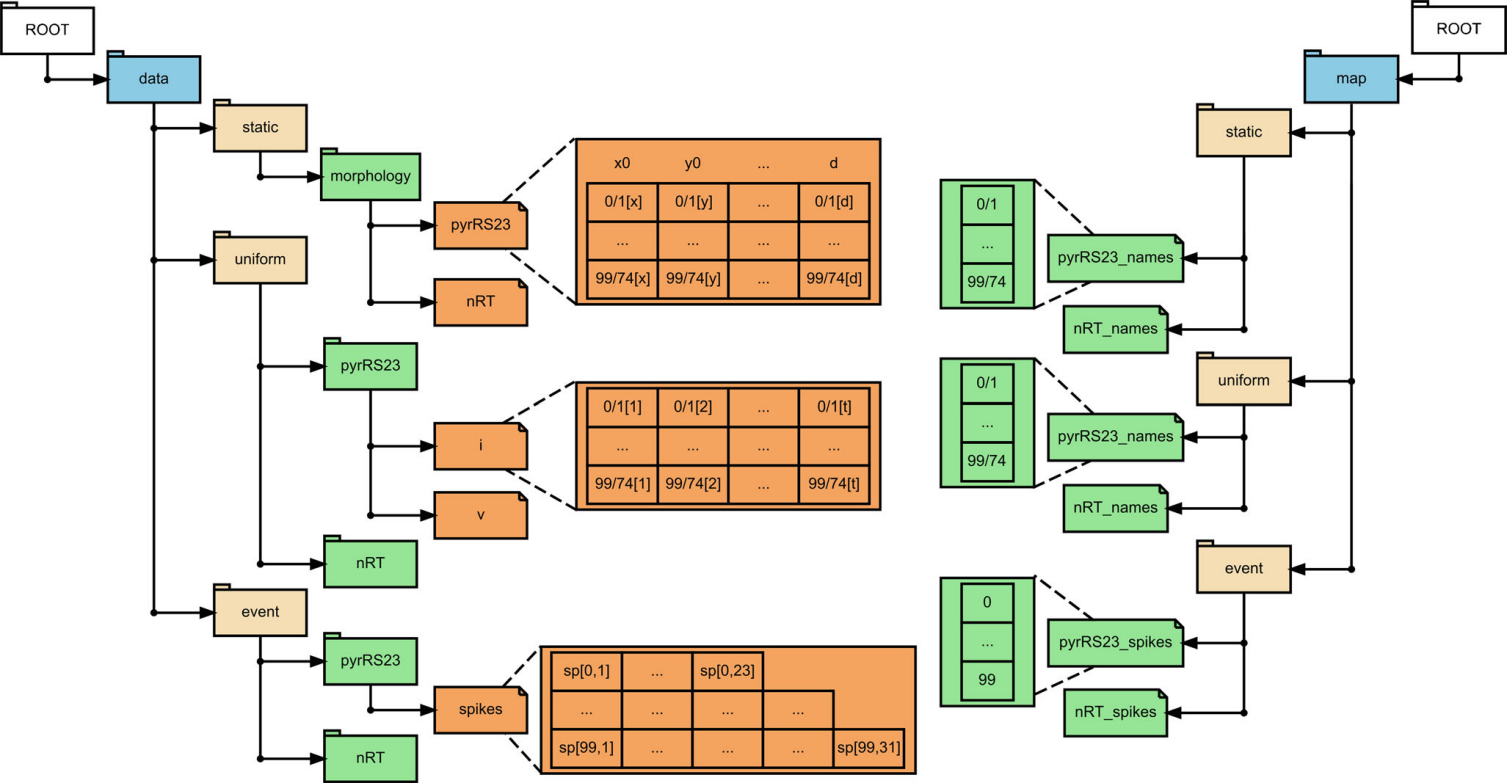
An illustration of the hierarchical storage of data in NSDF for an example subset of two cell populations from the down-scaled model. Note that the datasets we provide include data from all the cell populations. The *left side* indicates the paths to the data arrays under the data. The unique id’s of the segments or cell names on the *right* are stored as arrays under map. For instance, the morphology information for pyramidal regular spiking layer 2/3 cells is stored as a compound array at data/static/morphology/pyrRS23. Each row in this compound array is a segment whose unique id is located in the respective row of the /map/static/pyrRS23_names array. Here, the columns as indicated, *x*0,*y*0,...,*d* compose the header for the compound array; correspond to the proximal (*x*0,*y*0,*z*0) coordinate, the distal (*x*1,*y*1,*z*1) coordinate and the diameter *d* of the segment. The connection between the arrays in data/ and map/ is given by the HDF5 Dimension Scales according to the NSDF specification. Likewise, data/uniform/pyrRS23/i contains transmembrane currents through each segment (rows) over time (columns) and map/uniform/pyrRS23_names has the respective segment ids. Similarly, data/event/pyrRS23/spikes is a variable length array where each row corresponds to a cell and has the instances when it fired. The units for all the arrays are attached as the corresponding array attributes

Each dataset includes the following data: 1) morphology information i.e., segment geometry and position, stored as HDF5 compound arrays under data/static/morphology/ of the NSDF file; 2) spike times (1–24 in Table 2) and/or input spikes (17–20 and 25–28 in Table 2) stored as variable length arrays stored under data/event; and 3) transmembrane currents and membrane potentials stored in data/uniform/pop_name. For some datasets (24–28 in Table 2) individual ionic current contributions to transmembrane currents are also included here. The abbreviation used here for pop_name are from Table 1. For instance, the total transmembrane currents for all the pyramidal regular spiking layer 2/3 cells are located in a 2D array at data/uniform/pyrRS23/i. In these 2D arrays, the rows correspond to the unique segment id (for arrays in data/static and data/uniform), or the cell name (for arrays in data/event). These unique id’s are stored as lists in map. The connection between the arrays in data and map is via the HDF5 standard Dimension Scales specification as per NSDF. For example, the array in data/static/morphology/pyrRS23 are row-wise mapped onto the elements in map/static/pyrRS23_names using Dimension Scales, and likewise /data/uniform/pyrRS23/i, and /data/event/pyrRS23/spikes to map/uniform/
pyrRS23_names and map/event/pyrRS23_spikes, respectively. This organization is illustrated in Fig. 2. For the sake of simplicity, this figure shows the NSDF file architecture only for the down-scaled version of the model for two cell populations. The units for the arrays are included as array attributes according to the NSDF specification, for example /data/uniform/pyrRS23/i array attribute for “unit” is “nA”.

**Table 2:** An overview of the datasets provided

No.	Simulation type	Size (%)	Stop (ms)	Stim. delay (ms)	Stim. duration (ms)	Depol pop5 (nA)	Depol pop6 (nA)
1	Oscillations 200Hz	100	700	100	600	1	0.75
2	Oscillations 100Hz	100	700	100	600	1	0.75
3	Oscillations 50Hz	100	700	100	600	1	0.75
4	Oscillations 25Hz	100	700	100	600	1	0.75
5	Oscillations 12.5Hz	100	690	100	600	1	0.75
6	Oscillations 8Hz	100	700	100	600	1	0.75
7	Oscillations 4Hz	100	700	100	600	1	0.75
8	Oscillations 2Hz	100	700	100	600	1	0.75
9	Oscillations 200Hz no depol	100	700	100	600	0	0
10	Oscillations 100Hz no depol	100	700	100	600	0	0
11	Oscillations 50Hz no depol	100	700	100	600	0	0
12	Oscillations 25Hz no depol	100	700	100	600	0	0
13	Oscillations 12.5Hz no depol	100	700	100	600	0	0
14	Oscillations 8Hz no depol	100	700	100	600	0	0
15	Oscillations 4Hz no depol	100	700	100	600	0	0
16	Oscillations 2Hz no depol	100	700	100	600	0	0
17	Oscillations 12.5Hz input pop23	100	700	100	600	1	0.75
18	Oscillations 12.5Hz input pop4	100	700	100	600	1	0.75
19	Oscillations 12.5Hz input pop5	100	700	100	600	1	0.75
20	Oscillations 12.5Hz input pop6	100	700	100	600	1	0.75
21	Oscillations 12.5Hz no input	100	700	100	600	1	0.75
22	Oscillations 12.5Hz input 20 % of TCR	100	700	100	600	1	0.75
23	Pulse stimulus	100	210	70	2	1	0.75
24	Pulse stimulus 10 % model	10	600	300	2	0.5	0.375
25	Pulse stimulus passive current	10	600	300	2	0.5	0.375
26	Pulse stimulus passive soma & axon	10	600	300	2	0.5	0.375
27	Pulse stimulus passive axon	10	600	300	2	0.5	0.375
28	Pulse stimulus blocked Na current	10	600	300	2	0.5	0.375

These are described in detail in Section “Datasets”. For all the datasets here, recordings begin at 0 ms. In the column *Size*, 100 % size corresponds of 3560 cells and 10 % size to 356 cells. Next columns define: *Stop* — the end of the recordings as well as the end of the simulation; *Stim. delay* — the beginning of the stimulus; *Stim. duration* — the duration of the stimulus. *Depol pop5* — a constant depolarization current injection to layer 5 pyramidal cell somas to depolarize these cells to fire, and likewise *Depol pop6* — a constant depolarization current injection to layer 6 pyramidal cell somas, for them to fire. The following abbreviations are applicable here: *depol* — depolarization current injection, *TCR* — thalamocortical relay cells, *pop23* — pyramidal cells in layer 2/3, *pop4* — pyramidal cells in layer 4, *pop5* — pyramidal cells in layer 5, *pop6* — pyramidal cells in layer 6, Na — Sodium

## Datasets

In total, we present 28 datasets as listed in Table 2, 22 of these are responses to oscillatory input currents of different

frequencies (oscillations), and the other 6 are responses to pulse stimulus (pulse).

### Datasets 1–16

A sinusoidal current

2 $$ I(t) = I_{\text{inj}} \sin (2 \pi f (t-t_{0})) {\Theta}(t-t_{0}),  $$

of amplitude *I*_inj_=2 nA was injected into all of the TCR cells *t*_0_=100 ms after the onset of simulation. This caused an oscillatory response in the cortex. The frequency of these stimuli was *f*=200,100,50,25,12.5,8,4 or 2 Hz in the different datasets. Here, Θ(*t*) is the Heaviside function. In datasets 1–8, during the simulation, the somas of layer 5 and layer 6 pyramidal cells were depolarized with 1 nA and 0.75 nA current, respectively. These data were used to study the meaning of independent components of current source density reconstructed from LFP (Głąbska et al. 2014). They were also used to validate the generalized Laminar Population Analysis (Głąbska et al. 2016). The activity of the network recorded in the dataset 5 (12.5Hz oscillatory stimulus with depolarized infragranular pyramids) is shown in Fig. 1 of Głąbska et al. (2016), while the dataset 2 is used in Figs. 2 and 5A of Głąbska et al. (2014).

Datasets 9–16 correspond to sets 1–8, except they were simulated without depolarization currents in infragranular pyramidal cells and are provided for comparison.

### Datasets 17–21

Generalized Laminar Population Analysis (Głąbska et al. 2016) uses LFP and MUA from laminar recordings to decompose network activity into physiologically meaningful components. This can be interpreted as contributions to extracellular activity arising from the cells active in a specific layer. To validate this method we started with the simulation leading to the dataset 5 (12.5 Hz oscillatory input with depolarized infragranular pyramids). We then disabled all the connections and used the spiking activity from individual populations in dataset 5 to activate the network. In this way we generated four datasets corresponding to inputs from pyramids in layer 2/3 (dataset 17), spiny stellate cells in layer 4 (18), pyramids in layer 5 (19) and pyramids in layer 6 (20). To get a baseline LFP, another dataset (21) was generated with no population input. The spiking activity and the LFPs obtained from these simulations are shown in Fig. 5 in Głąbska et al. (2016).

### Dataset 22

These are the data from a simulation similar to that of the dataset 5, except that only 20 % of the TCR cells received the oscillatory input, (Eq. (2)), here. This resulted in less correlated network activity.

### Dataset 23

To simulate the cortico-thalamic responses to a whisker deflection in a rodent we injected a constant current pulse of amplitude 3 nA for a duration of 2 ms into the TCR cells, 70 ms from the start of the simulation. Layer 5 and 6 pyramidal cells’ somas were depolarized with 1 and 0.75 nA currents, respectively, during the simulation. Such a stimulus caused a brief (about 5 milliseconds) activation of the TCR cells. The activity then propagates to the spiny stellate cells in layer 4, the deep basket interneurons in layers 5–6, and the nucleus reticularis in the thalamus. Then, the activation appears in the fast rhythmic bursting cells in layer 2/3 and several milliseconds later in tufted pyramidal intrinsic bursting and regular spiking cells in layer 5, pyramidal regular spiking cells and interneurons in layer 2/3. Finally, the stimulus reaches the rest of the cortical cells: nontufted pyramidal regular spiking neurons in layer 6 and the interneurons in layer 5/6. After 50 ms from the onset of the stimulus the network settles down again. In this case we used the whole model and recorded only the spike times, the membrane potential and the total transmembrane current in every segment. The simulationends at 180 ms when the evoked activity dies out. Figure 2 in Głąbska et al. (2014) shows the raster plot for this kind of simulation.

### Dataset 24

**Fig. 3:**
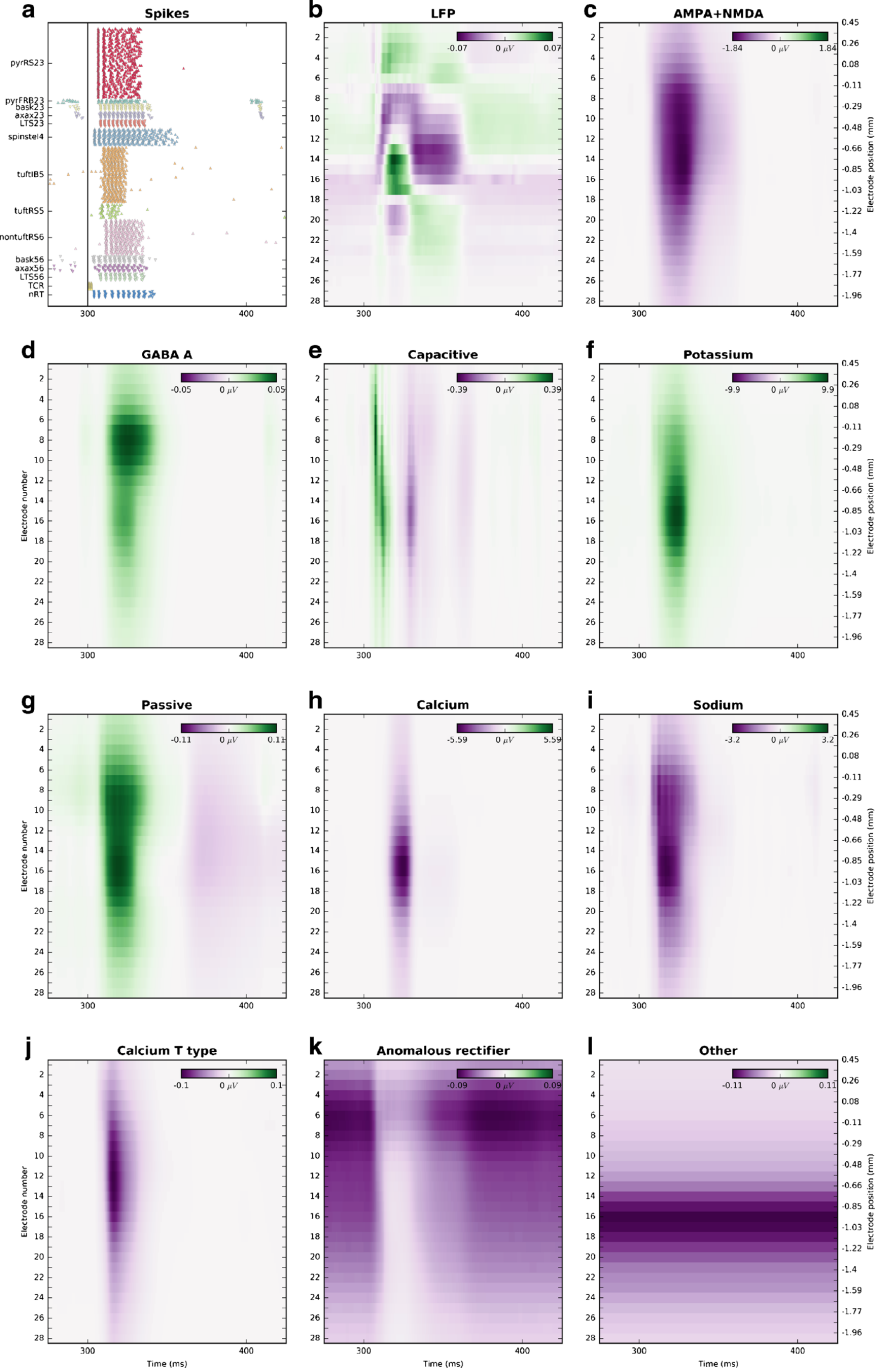
Dataset 24. **a** raster plot of the network activity. *Up and down pointing triangles* are excitatory and inhibitory neurons, respectively, the *black vertical line* shows the stimulus onset (**b**–**l**) contributions to the extracellular potentials as recorded by 28 electrodes with inter-electrode distance of 92.6 *μ* *m*, *left y-axis* shows electrode number, right y-axis shows depth; (**b**) LFP and contributions to LFP (extracellular potential filtered below 100 Hz using second order Butterworth filter) from specific currents, respectively: (**c**) NMDA + AMPA (**d**) GABA (**e**) capacitive, (**f**) potassium, (**g**) passive, (**h**) calcium, (**i**) sodium (**j**) two kinds of calcium low threshold T type currents not causing [Ca2+] influx (**k**) anomalous rectifier, (**l**) all other currents, such as ectopic currents and depolarizing currents. Note that the scales used in different panels differ to emphasize the individual contributions

This is similar to the dataset 23, however, only 10 % of the cells were used and layer 5 and layer 6 cells were depolarized by 0.5 nA and 0.375 nA respectively to obtain firing rates consistent with the full model. The stimulus was applied 300 ms from simulation onset after initial transient died out. In this dataset we recorded the sum of transmembrane currents, membrane potential, as well as different contributions to the transmembrane currents: current flowing through synapses GABA A, NMDA and AMPA, capacitive, passive, potassium, sodium, calcium, two kinds of calcium low threshold T type currents (not causing [Ca2+] influx), anomalous rectifier, and other currents (e.g. steady bias + ectopic currents). Figure 3 shows the raster plot from this simulation, the LFP (extracellular potential filtered below 100 Hz using second order Butterworth filter), and the components of the LFP which originate from the different types of recorded current sources.

### Datasets 25–28

**Fig. 4:**
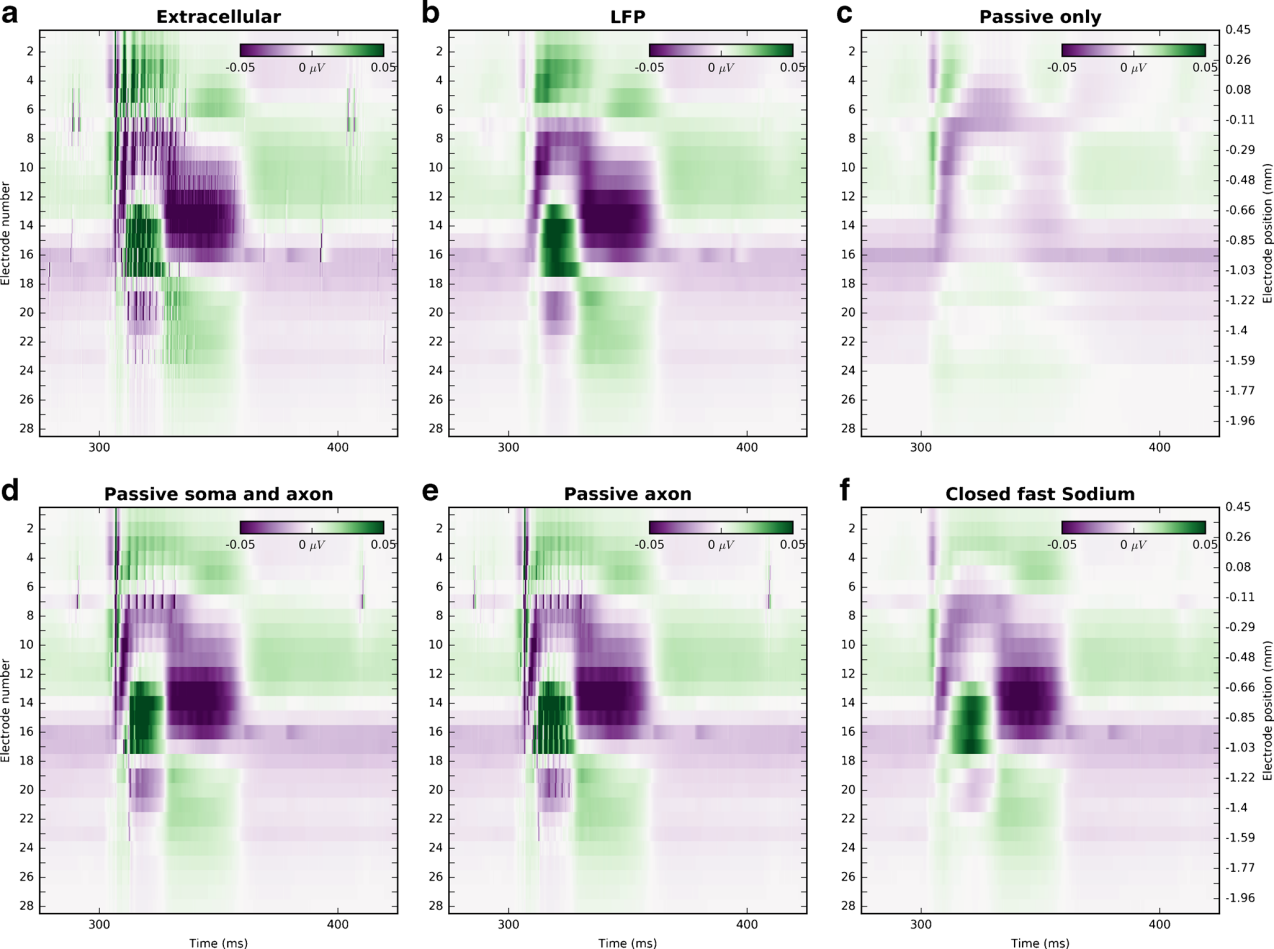
Potentials as recorded by 28 electrodes with inter-electrode distance of 92.6 *μ* *m* distance, *left y-axis* is electrode number, *right y-axis* is its depth (**a**) Extracellular potential from dataset 24 (**b**) the same as A but filtered below 100 Hz (LFP) using second order Butterworth filter. The following panels show not filtered extracellular potential from data sets (**c**) 25 (**d**) 26 (**e**) 27 (**f**) 28. The stripy structures visible in panel A (unfiltered extracellular potential) are the extracellular signatures of spiking (not shown but can be visualized with the provided data)

The simulations in which these data were obtained are all similar to those leading to the dataset 24. They were obtained to investigate how the transmembrane currents through active channels are reflected in extracellular recordings. For this we performed simulations with active channels turned off in: every segment (dataset 25), in somas and axons (26), only in axons (27), or we turned off only fast sodium currents in every segment (28). Since closing these channels silenced the network, we stimulated the system with spikes recorded in dataset 24. Thus these datasets can be used to identify the differences in LFP where the same synaptic stimuli are provided while different combinations of the active channels are closed. The LFP resulting from these simulations are presented in Fig. 4. The stripy structures visible in panel A (unfiltered extracellular potential) are the extracellular signatures of spiking (not shown but can be visualized with the provided data).

## Discussion

Traub’s model of the thalamo-cortical column (Traub et al. 2005) is one of the largest and most popular conductance based multi-compartmental models that is publicly available. It serves as an important benchmark for every simulator and its established position is apparent by the fact that the original FORTRAN model has been translated into NEURON, MOOSE, and NeuroML versions. This model has its limitations, some of which were discussed in the original paper (see Discussion in Traub et al. 2005), some were discovered later on Gleeson et al. (2013), some are consequences of translations to new platforms (see Fig. 10, Gleeson et al. 2010). A number of difficulties with different translations of this model were documented and tested by the research community outside the laboratories where the model was originally developed (Gleeson et al. 2013). This model is a good starting point for modeling thalamo-cortical system, as it illustrates the complexity of such large scale modeling studies.

Here we presented 28 simulation datasets of Traub’s thalamo-cortical model. In all the datasets positions of the cells, cell morphologies, membrane voltage potential, transmembrane currents, and the spiking information are provided. In three datasets, 22–24, we also provide contributions from individual channel types to the transmembrane current.

Some of these datasets were used or are equivalent to those used in our previous research. In particular, datasets 1–8 and 23 or equivalent were used in a study of physiological meaning of independent components of current source density reconstructed from LFPs (Głąbska et al. 2014). In a study of the effects of physical and geometrical properties of a cortical slice in saline on extracellular potentials recorded with multielectrode arrays and for reconstruction of current source density in such a setup (Ness et al. 2015) we used data from the dataset 3. We also used the datasets 1–8 and 17–21 to validate the generalized Laminar Population Analysis (Głąbska et al. 2016).

The data provided here can be used to test and compare different spike detection algorithms, or to test the validity of hybrid methods for calculating the extracellular potentials, where combinations of point neurons with single multicompartmental neuron models are used. These data may also be used to investigate the relationship between the actual transmembrane currents and the reconstructed CSD (e.g. Głąbska et al. 2014, Fig. 4), serve as a ground truth for analysis methods based on the reconstructed CSD, or used to compare different methods of current source density estimations using the extracellular potentials they generate.

Due to the latest advancements in microelectrode technology, sophisticated configurations of electrode placements are possible. With the help of the provided data it is possible to model simultaneous extracellular potential recordings in these configurations. For example, it is possible to compare the recordings of a laminar probe placed next to the cortical column, on a 2D electrode grid of multishank electrode, but one could also investigate contributions to ECoG or EEG. However, this would require more complex models of field propagation taking into account geometry and conductivity of the cranium, scull and scalp. We hope that the availability of these data would facilitate understanding of the relationship between the network activity and measurement, would help with the interpretation of the results of specific analytic methods and lead to new insights.

The data are provided in NSDF (Ray et al. 2016), a well documented subspecification of HDF5, developed for storage of the data from simulations. Any visualization or analysis tools developed to support HDF5 in general, such as HDFView, will be applicable to these datasets.

The first 16 datasets show responses of the network to 8 different stimuli in two network states (with extra depolarization of infragranular pyramids and without). To facilitate validation of more involved methods of data analysis we performed additional simulations for injection of 12.5 Hz sinusoidal current. Datasets 17–21 attempt to uncover what part of the whole network activity is driven by specific populations (Głąbska et al. 2016). These contributions cannot be obtained experimentally, as they require the use of spikes from a population functioning in a fully connected network to drive the network of disconnected cells. In experiment, even if we were able to silence connections within the network except from those coming from a particular population, system activity would change, so the network response would also change.

Driving all the TCR cells with the same oscillatory stimulus imposes strong correlations on the network activity. Since the correlations in spiking activity and input currents affect the extracellular potential power spectrum and the spread of the signal (Łęski et al. 2013), we generated additional datasets where only 20 % of the TCR cells were driven (dataset 22). That was enough to observe a response in the whole network which was less correlated.

Datasets 24–28, can be used to test different hypotheses and interpretations of LFP on the model data, study the relation of LFP to postsynaptic currents, identification of currents contributing the most to the LFP, relation between spiking and the LFP, etc. To facilitate investigations of such questions, we recorded all of the transmembrane currents separately. Since the size of these datasets is substantial, we decided to run a down-scaled version of the Traub model with only 10 % of the cells. Interestingly, the largest contributions to the extracellular potential comes from excitatory synaptic currents as well as from the active currents, and they are an order of magnitude larger than the final LFP, see Fig. 3. However, due to extensive but nontrivial cancellations, all the currents, including passive and capacitive currents, contribute significantly to the LFP, which makes interpretation of the LFP signal a challenging task. In the datasets 25–28 we prevent spiking behavior of the network using different approaches, (for details see Section “Datasets” or Table 1), but we provided the same synaptic stimulus as in the dataset 24. In every case the high frequency signal in extracellular potential was reduced. With the exception of the case where we blocked all the active channels (dataset 25, Fig. 4 c), the low frequency part of the potential remained similar as in the original simulation (dataset 24, Fig. 4 b). This is a strong evidence that the LFP is evoked by synaptic activity, while at the same time we see that, at least in these model data, active channels in the dendrites play a critical role in setting up the LFP signal.

To obtain the data presented here, powerful computational resources are needed, that are not easily accessible to every researcher. Even if these computational resources are available, it is impractical and wasteful to duplicate efforts to set up and run such large simulations for a single laboratory use. For example, we performed the computations on the IBM Blue Gene Q computer at the Interdisciplinary Center of Modeling, University of Warsaw, using 64 nodes, each equipped with 16 cores and 16 GB of memory. The wait time to avail of these resources was typically 1–3 days and the simulations lasted typically 8–10 hours. We hope that if the interest in this model sustains and other researchers perform new simulations with parameters different from those listed here, a more extensive collection of ground truth data will be established, to further facilitate studies of relations between system internals and measurements, and for validation of complex methods of data analysis, called for by the results of the present day experiments. The data provided here were generated with public resources and the results of such an endeavor rightfully belong to the community at large.

## Information Sharing Statement

The complete collection of datasets provided here is available at 10.18150/repod.6394793, hosted by RepOD (RRID:SCR_014697). These files are available under the Open Database License (ODbL 1.0 license). We provide the NEURON (RRID:SCR_005393) code used to generate these datasets at https://github.com/Neuroinflab/Thalamocortical. We also provide here Python scripts to convert IBM Blue Gene Q computer output of NEURON to NSDF file format, and some Python based analysis scripts to generate LFP for 1, 2, and 3 dimensional electrode layouts, raster plots, NEO objects, etc, for an NSDF file. These scripts are available under the GNU GPL 3.0 License.

## Footnotes

1 LFP came about as the acronym of ’local field potential’, which, as we know today, is a misnomer. It was shown by many (see Buzsáki et al. 2012; Einevoll et al. 2013 for an overview) including us Łęski et al. (2007) and Hunt et al. (2011) that due to the long-range nature of the electric field the same sources are visible in the rat’s brain on distances of the order of the whole brain, which makes LFP a very *non-local quantity*. This is why we suggest to drop the name local field potential and read LFP as ’low frequency part’ of extracellular potential, which is the definition of LFP.
